# The Leukocyte Immunoglobulin-Like Receptor Family Member LILRB5 Binds to HLA-Class I Heavy Chains

**DOI:** 10.1371/journal.pone.0129063

**Published:** 2015-06-22

**Authors:** Zhiyong Zhang, Hiroko Hatano, Jacqueline Shaw, Marloes Olde Nordkamp, Guosheng Jiang, Demin Li, Simon Kollnberger

**Affiliations:** 1 Nuffield Department of Orthopaedics, Rheumatology and Musculoskeletal Sciences, Botnar Research Centre, University of Oxford, Oxford, United Kingdom; 2 RDM Clinical Laboratory Sciences, John Radcliffe Hospital, University of Oxford, Oxford, United Kingdom; 3 Department of Hemato-Oncology, Institute of Basic Medicine, Shandong Academy of Medical Sciences, Shandong Jinan, Shandong, Peoples Republic of China; University of East London, UNITED KINGDOM

## Abstract

**Objective:**

The leukocyte immunoglobulin-like receptor (LILR) family includes inhibitory and stimulatory members which bind to classical and non-classical HLA-class I. The ligands for many LILR including LILRB5 have not yet been identified.

**Methods:**

We generated C-terminal eGFP and N-terminal FLAG-tagged fusion constructs for monitoring LILR expression. We screened for LILR binding to HLA-class I by tetramer staining of 293T cells transfected with LILRA1, A4, A5 A6 and LILRB2 and LILRB5. We also studied HLA class I tetramer binding to LILRB5 on peripheral monocyte cells. LILRB5 binding to HLA-class I heavy chains was confirmed by co-immunoprecipitation.

**Results:**

HLA-B27 (B27) free heavy chain (FHC) dimer but not other HLA-class I stained LILRB5-transfected 293T cells. B27 dimer binding to LILRB5 was blocked with the class I heavy chain antibody HC10 and anti-LILRB5 antisera. B27 dimers also bound to LILRB5 on peripheral monocytes. HLA-B7 and B27 heavy chains co-immunoprecipitated with LILRB5 in transduced B and rat basophil RBL cell lines.

**Conclusions:**

Our findings show that class I free heavy chains are ligands for LILRB5. The unique binding specificity of LILRB5 for HLA-class I heavy chains probably results from differences in the D1 and D2 immunoglobulin-like binding domains which are distinct from other LILR which bind to β2m-associated HLA-class I.

## Introduction

The leukocyte immunoglobulin-like receptor (LILR) family includes both inhibitory and stimulatory receptor members that regulate immune responses (reviewed in[1]). Activating (LILRA) or inhibitory (LILRB) receptors differ in residues within their transmembrane and cytoplasmic domains, while extracellular membrane distal D1 and D2 immunoglobulin-like (Ig) domains bind to ligand. The inhibitory receptors incorporate long cytoplasmic tails with ITIM motifs which are phosphorylated upon cell activation and receptor ligation and inhibit leukocyte activation through SHP phosphatase recruitment. The stimulatory receptors have a shorter tail and interact with ITAM incorporating adaptor molecules to activate immune cells.

The LILRA1, A4, A5 and A6 and LILRB2 molecules in this study are expressed by cells of the myelomonocytic lineage [1]. LILRB2 is also expressed by human hematopoietic stem cells [2]. LILRB5 transcripts have been detected in natural killer (NK) lymphocytes [3], mast cells[4], macrophages and osteoclasts[5]. LILRB5 has also been previously reported to be expressed intracellularly in mature human mast cells [4].

LILRA1 binds to both classical β2m and peptide-associated HLA-B27 and HLA-B27 free heavy chain dimers (termed B27_2_ [6]). LILRB2 binds to classical β2m and peptide-associated HLA-class I, non-canonical HLA-G, HLA-B27 free heavy chain (FHC) dimers and other HLA-class free heavy chains [7–10]. Ligands for LILRB5 have not previously been identified.

LILR receptors incorporate 2–4 extracellular Ig-like domains termed D1, D2, D3 and D4. The membrane distal D1 and D2 domains form the domains for binding to HLA-class I ligands for receptors such as LILRB1 and LILRB2. The D3 and D4 domains form a stalk region, enabling some LILR receptors such as LILRB2 to bind to class I ligands both in *cis* (on the same cell) and in *trans* orientations [11]. Recently it has been shown that both the D1 and D4 domains of LILRB2 are necessary for binding to the non-HLA ligand angiopoetin-like protein 2 (Angtpl2) [12].

HLA-B27 is strongly associated with development of the spondyloarthritides (SpA), typified by ankylosing spondylitis, where 94% express HLA-B27 [13]. HLA-B27 is expressed at the cell surface both as classical β2m-associated and cysteine-67 dependent disulphide-linked β2m-free heavy chain dimer forms[14]. Increased expression of B27 dimers has been implicated in SpA disease [15]. The differential binding of B27 dimers and classical β2m-associated HLA-B27 to LILR and other immune receptors has been proposed to be involved in the pathogenesis of SpA [16].

Here we use tetramer staining to screen a series of LILR expression constructs for binding to HLA-class I and identify LILRB5 as a new receptor for HLA-class I heavy chains. We further characterise binding of this LILR member to HLA class I biochemically and by FACS staining with B27 dimer tetramers of the LILRB5 receptor on peripheral monocytes.

## Materials and Methods

### Cell lines and antibodies

LBL.721.221 cells and LBL.721.220 (abbreviated to 221 and 220 [17]) transfected with HLA-B*2705 and HLA-B*0701 have been described previously [18, 19]. 221 and 220 cells were transduced with PHR-SIN lentivirus encoding LILRB2 or LILRB5 as described in[10]. RBL cells were transduced with PHR-SIN lentivirus encoding HLA-B*27:05 and HLA-B*0701 [20] in addition to lentivirus encoding LILRB2 and LILRB5

W6/32, ME1 and HC10 mAbs, originally purchased from the European Collection of Cell Cultures were produced and purified in-house. M2 anti-FLAG mAb (allophycocyanin conjugated for FACS staining) was purchased from R&D Systems. IgG1, IgG2a mAbs were purchased from eBioscience and APC-conjugated and PE-conjugated Goat anti-mouse Igs were from Biolegend. Goat anti-LILRB5 (anti-hLIR8) antiserum and control normal goat antiserum (NGS) were obtained from R and D systems. This antiserum has been raised against LILRB5 peptides and has minimal cross-reactivity against other LILR family members including LILRB1 and LILRB2.

Peripheral blood mononuclear cells (PBMC) from healthy donors were isolated as previously described [21].

### Generation of plasmid constructs for expression of innate immune receptors

CDNAs encoding FLAG-tagged LILRA1. A4, A5, A6, B2 and B5 were generated by PCR using Phusion High–Fidelity DNA Polymerase (New England Biolabs) and cloned into pEGFP to generate expression constructs for N-terminal FLAG and C-terminal eGFP tagged fusion proteins (primers are listed in S1 Table). Subsequently plasmids were used for transfection of 293T cells using GeneJuice according to the manufacturers instructions (Novogen, CA, USA). Expressed fusion proteins were detected by fluorescent microscopy and FACS staining with anti-FLAG MAb.

### FACS staining with HLA-class I tetramers

HLA-B27 dimer and HLA-class I tetramers were generated and used for FACS staining of transfected 293T cells and monocytes as previously described [21, 22]. Tetramer staining was blocked with HC10 MAb as described in [21, 22]. The HLA-B*0701 tetramers with the Nef RL10 peptide were a generous gift from Professor Tao Dong.

### Lentiviral transduction of cell lines with LILRB5

A pHR-SIN lentiviral vector was constructed incorporating LILRA5, LILRB5 with an N terminal FLAG tag and C terminal eGFP tag. Generation of lentivirus encoding HA-tagged LILRB2 has been described previously. The primers for generation of LILR expression constructs are listed in S1 Table.

LILRB5 lentivirus were generated by transfection of 293T cells with pHR-SIN-LILRB5, the viral envelope (VSV-G) and polymerase (GAG) using Genejuice (Novagen, Merck, CA. USA). Supernatants containing lentivirus were harvested on day 3 and used to stably transduce 221, 220, and RBL cells as described in [19]. LILRB5 expression was detected by immunofluorescence microscopy and FACS staining with anti-FLAG antibody.

Parental 221 cells or 221 cells transfected with HLA-B27, HLA-A3, and HLA-B35 and 220 cells transfected with HLA-B27 were transduced with tagged LILRB5. Alternatively RBL cells were transduced with LILRB5 and HLA-B7 or B27 lentivirus (previously described in [10]). HLA-class I expression was monitored by flow cytometry with W6/32, HC10, and ME1 antibodies (produced in house) and APC conjugated Goat anti-mouse Igs (Biolegend).

### Immunoprecipitations

Cell lysates for immunoprecipitations and western blots of immunoprecipitates with HC10 antibody were prepared and performed as described in [18]. Immunoprecipitates were resolved by SDS-PAGE under reducing conditions. Immunoprecipitations with HC10 MAb, rabbit anti-FLAG (Sigma, UK, Ltd) or isotype control antibodies (IgG2a, Biolegend) were performed with anti-mouse Igs or Protein G dynalbeads following the manufacturers instructions (Dynalbeads UK Ltd). HRP-conjugated anti FLAG antibody (M2) antibody for western blots was obtained from Sigma and used at the manufacturer’s recommended dilution.

### Ethical Approval

Ethical approval for obtaining blood samples from healthy controls for this study was obtained from Oxfordshire Research Ethics Committee (REC) C as part of the study "Immune function in inflammatory arthritis". REC reference number: 06/Q1606/139. In procedures approved by Oxfordshire REC C, written informed consent was obtained from all healthy donors and a written dated record of the time and purpose of use of samples maintained in laboratory records.

## Results

### HLA-B27 free heavy chain dimers bind to LILRA1, LILRB2 and LILRB5 but not LILRA4, A5 and A6

**Fig 1:**
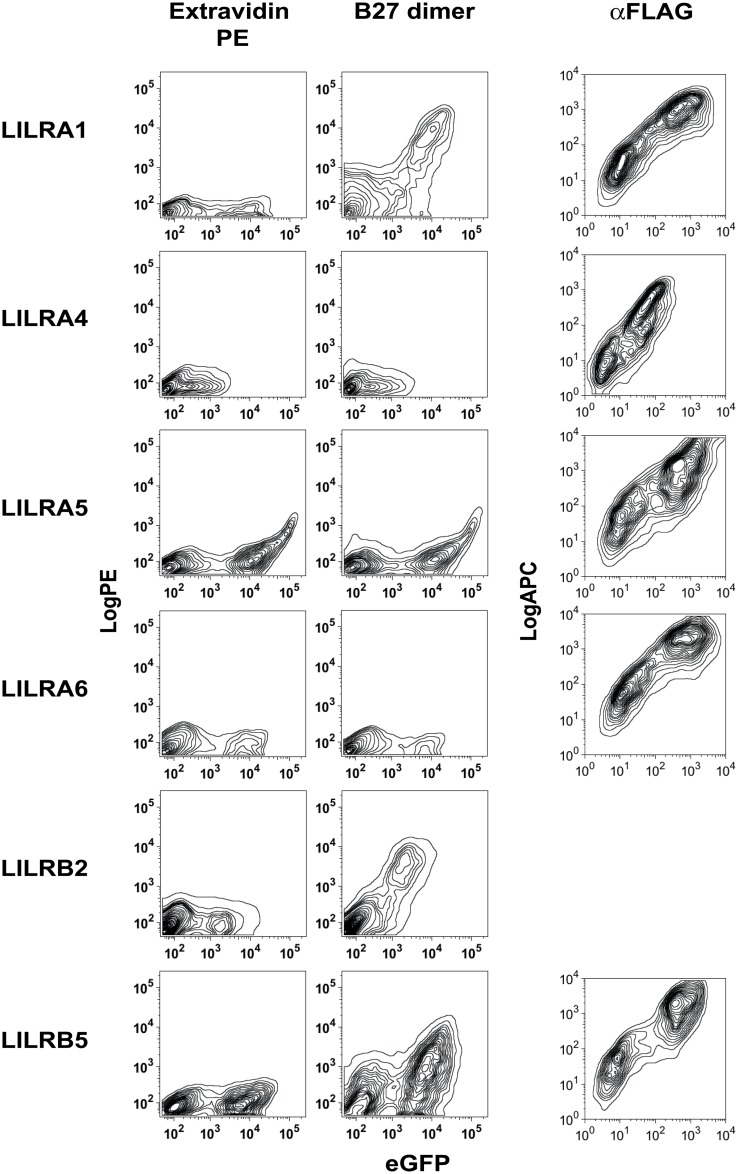
HLA-B27 free heavy chain dimers bind to LILRB5. FACS staining of 293 T cells transfected with eGFP and FLAG-tagged constructs of LILRA1, LILRA4, LILRA5, LILRA6, LILRB2 and LILRB5 stained with Extravidin-PE (left panels) or Extravidin PE-conjugated HLA-B27 free heavy chain dimer tetramers (centre panels). FACS plots show PE fluorescence from tetramer or Extravidin staining plotted against eGFP expression of each of the fusion experiments. (Right panels) FACS staining of 293 T cells transfected with eGFP and FLAG-tagged constructs of LILRA1, LILRA4, LILRA5, LILRA6, and LILRB5 stained with allophycocyanin (APC) conjugated anti-FLAG antibody. FACS plots show APC fluorescence from anti-FLAG staining plotted against eGFP expression of each of the fusion constructs. Representative FACS stain from 1 of 4 independent experiments.

We and others have previously reported that HLA-B27 β2microglobulin (B27) free heavy chain dimers bind to the leukocyte immunoglobulin-like receptors LILRA1 and LILRB2 [9, 10, 21]. We extended these studies to investigate binding of B27 dimers to other LILR members. 293T cells were transfected with FLAG and eGFP-tagged B5, A1, A4, A5 and A6 or HA and eGFP tagged LILRB2 expression constructs and stained with extravidin PE or extravidin PE-conjugated B27 dimer tetramer (Fig 1). Good levels of expression of all constructs used in this study were observed as indicated by detection of eGFP fluorescence by FACS (Fig 1). B27 dimer tetramers stained LILRA1 and LILRB2 transfected cells in agreement with previous results [9, 21]. B27 dimer tetramers also stained LILRB5 transfected cells (Fig 1). We have previously shown cell surface expression of HA and eGFP tagged LILRB2 in transfected 293 T cells [10]. LILRA1, A4, A5 and A6 and LILRB5 constructs were expressed at the surface of transfected cells as assessed by staining with anti-FLAG antibody (Fig 1).

Although LILRA4, LILRA5 and LILRA6 were highly expressed in 293T cells, as assessed by staining with anti-FLAG antibody and eGFP fluorescence, they did not bind to B27 dimers (Fig 1).

### LILRB5 binds specifically to HLA-B27 dimers but does not bind to β2m and peptide associated HLA-A3, HLA-B7 and HLA-B27 heterodimers

**Fig 2:**
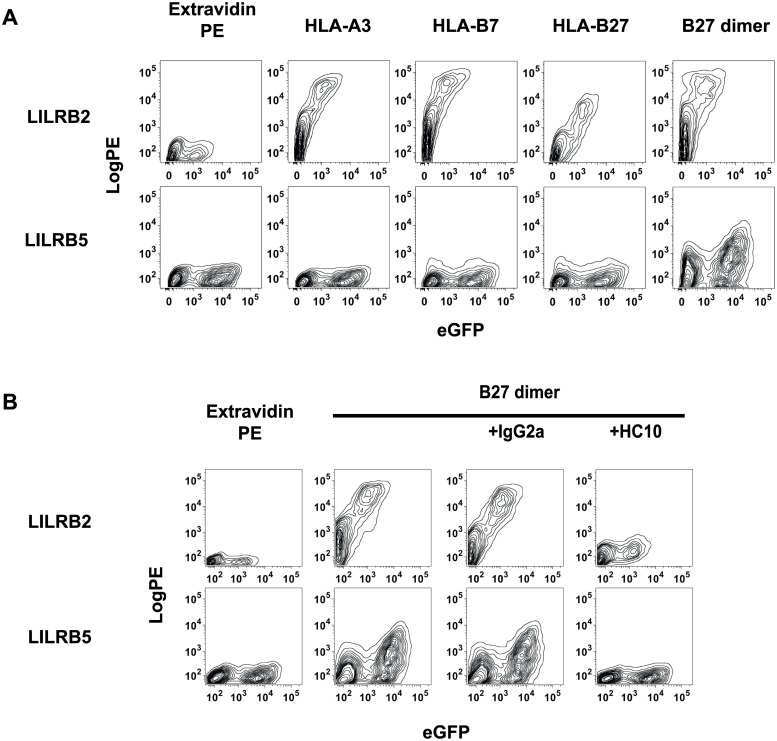
LILRB5 binds specifically to HLA-B27 free heavy chain dimers but does not bind to β2m and peptide associated HLA-A3, HLA-B7 and HLA-B27 heterodimers A. FACS staining of 293 T cells transfected with eGFP and FLAG-tagged constructs of LILRB2 and LILRB5 and stained with Extravidin-PE, orExtravidin-PE conjugated tetramers of HLA-A3, HLA-B7, and HLA-B27 heterodimer or HLA-B27 free heavy chain (FHC) dimers. FACS plots show PE fluorescence from tetramer or Extravidin staining plotted against eGFP expression of each of the fusion experiments. Representative FACS stain from 1 of 3 independent experiments **B**. FACS staining of LILRB5 transfected 293T cells with HLA-B27 FHC dimer tetramer or Extravidin PE with or without isotype control antibody (IgG2a) or free heavy chain antibody HC10 as indicated. FACS plots show PE fluorescence from tetramer or Extravidin staining plotted against eGFP expression of each of the fusion experiments. Representative FACS stain from 1 of 3 independent experiments.

Both B27 dimers and β2microglobulin-associated HLA-class I heterodimers bind to LILRB2. It is unknown whether β2microglobulin-associated HLA-class I bind to LILRB5. Thus, we next stained LILRB2-, and LILRB5-transfected 293T cells with extravidin PE, or HLA-A3, HLA-B7, and HLA-B27 heterodimer and B27 homodimer tetramers. LILRB2 transfected 293T cells stained with both heterodimer and homodimer tetramers stained LILRB5 transfected 293T cells (Fig 2A).

We have previously shown that the HLA-class I heavy chain antibody HC10 blocks B27 dimer binding to LILRB2 [10]. We next determined whether HC10 antibody could also block binding of B27 dimers to LILRB5. Pre-incubation with the HLA-class I heavy chain antibody HC10 blocked B27 dimer tetramer staining of LILRB5 transfected 293T cells (Fig 2B). Isotype control antibody (IgG2a) had no effect on B27 dimer binding to LILRB5 (Fig 2B).

**Fig 3:**
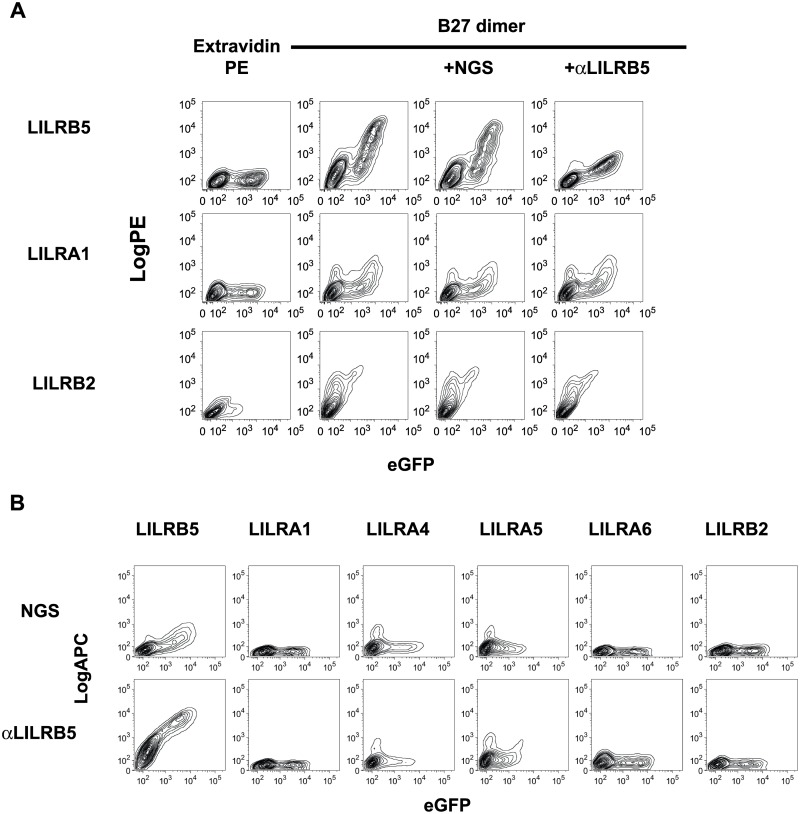
B27 dimer binding to LILRB5 is inhibited by LILRB5 specific antiserum. **A.** FACS staining of LILRB5, LILRA1 and B2 transfected 293T cells with HLA-B27 FHC dimer tetramer or Extravidin PE with or without isotype control antisera (ISOT) or anti-LILRB5 antisera (αLILRB5) as indicated. FACS plots show PE fluorescence from tetramer or Extravidin staining plotted against eGFP expression of each of the respective fusion constructs. Representative FACS stain from 1 of 3 independent experiments. **B.** Representative FACS staining of 293T cells transfected with the indicated LILR constructs and stained with anti-LILRB5 or normal goat antisera (NGS). Representative stain from 1 of 3 independent experiments.

We next addressed to what extent anti-LILRB5 antisera could specifically inhibit B27 dimer binding to LILRB5 transfected cells. Preincubation of LILRB5-transfected cells with anti-LILRB5 antisera inhibited B27 dimer staining of LILRB5-transfected 293T cells but did not affect B27 dimer staining of 293T cells transfected with LILRB2 or LILRA1 (Fig 3A). Control normal goat antiserum (NGS) had no effect on B27 dimer tetramer staining of LILRB5 transfected 293T cells (Fig 3A).

We tested the specificity of the LILRB5 antiserum by FACS staining 293T cells transfected with different LILR. LILRB5 antiserum stained 293T cells transfected with LILRB5 but did not stain any of the other LILR in this study (Fig 3B).

### Sequence alignment of the D1 and D2 binding domains of LILRB1, LILRB2 and LILRB5 reveal a possible basis for binding differences with HLA class I

LILRB1 binds to β2m-associated HLA-class I. By contrast, LILRB2 binds to both β2m-associated HLA-class I and β2m-free heavy chain forms of HLA-class I including HLA-B27 heavy chain dimers. Based on sequence similarity LILRB5 has been suggested not to bind to β2m-associated HLA class I which is consistent with our results. Our results indicated that HLA-B27 dimer tetramers but not β2m-associated HLA-class I tetramers bound to LILRB5.

We aligned the D1 and D2 binding domains of LILRB1, LILRB2 and LILRB5 and LILRA4, 5 and 6 in order to identify differences and similarities in potential contact residues in these regions which have previously been shown to contact HLA-class I (S1 Fig). Amino acids in LILRB1 and LILRB2 which have been identified for binding β2microglobulin-associated HLA class I are indicated with a hatch or asterisk respectively. Amino acids are coloured based on degree of homology to the consensus sequence of the LILR members studied in this alignment [7, 23, 24]. Arrows indicate the positions of β strands.

Although LILRB5 shared 67% sequence similarity with LILRB2, only 5 of the amino acids that have been shown to bind to classical β2m-associated HLA class I in LILRB2 are the same in LILRB5 (S1 Fig).

### Surface expression of LILRB5 is primarily detected on peripheral monocyte cells

**Fig 4:**
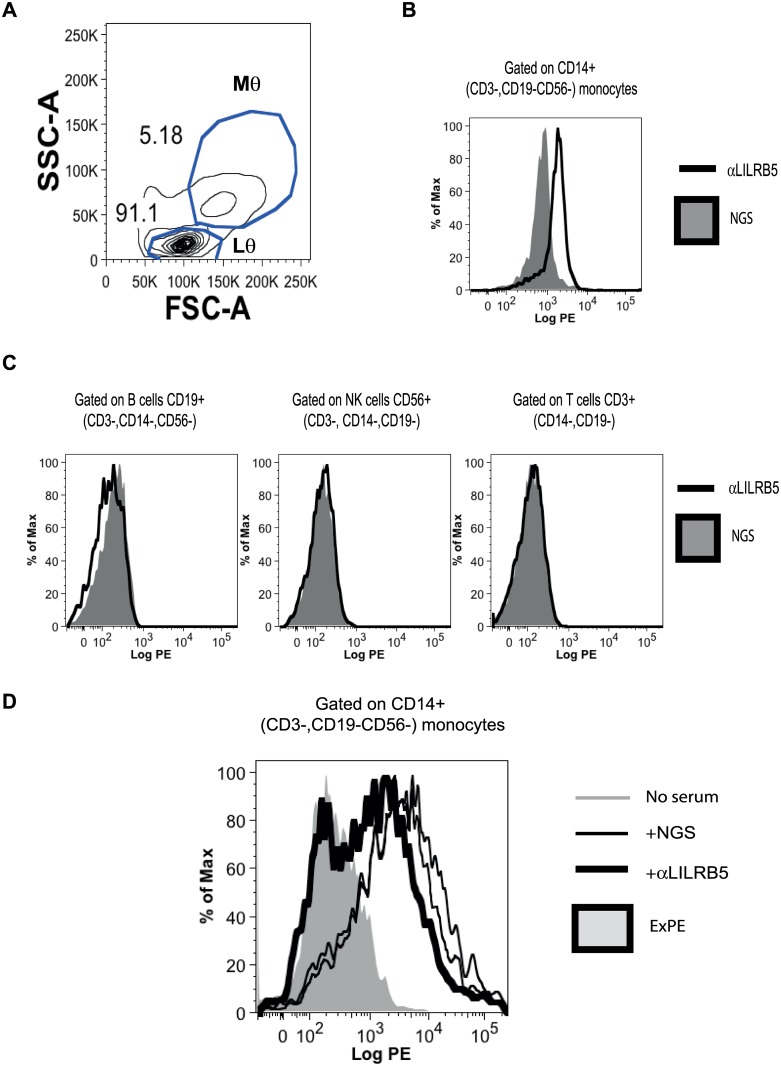
LILRB5 is expressed on the surface of peripheral monocytes. B27 heavy chain dimers bind to monocyte LILRB5. **A.** Forward scatter (FSC-A) and side scatter (SSC-A) FACS plot of peripheral blood mononuclear cells (PBMC) showing gating strategy for monocytes (Mθ) and lymphocyte (Lθ) populations. The relative proportions of monocyte and lymphocyte populations are indicated to the side of each gate. **B.** FACS staining of peripheral CD14+ (CD3-, CD19-, CD56-) monocytes with anti-LILRB5 antiserum (bold line) or normal goat serum (NGS, shaded histogram). **C.** FACS staining of (left panel) CD19+ (CD3-,CD14-CD56-) peripheral B, (centre panel) CD56+ (CD3-,CD14-,CD19-) natural killer (NK) or (right panel) CD3+ (CD14-,CD19-) T cell populations with anti-LILRB5 antiserum (bold line) or NGS (shaded histogram). Representative staining from 1 of 3 independent experiments. **D.** B27 dimer tetramer staining of peripheral monocytes without antiserum (dashed line) or in the presence of NGS (light line) or LILRB5 antiserum (bold line). FACS staining with extravidin PE (ExPE, shaded histogram) is included as a background staining control. The respective geometric mean fluorescent intensities (MFI) for staining without antiserum, with NGS and with anti-LILRB5 antiserum were 2498, 2827 and 881 respectively. The geometric MFI for staining with extravidin PE was 236. Representative staining from 1 of 3 independent experiments.

We next stained peripheral blood mononuclear cells with anti-LILRB5 or control antisera to determine surface expression of this receptor by lymphocyte and monocyte populations by multiparameter flow cytometry (Fig 4). Lymphocyte and monocyte gates for flow cytometry were drawn on the basis of forward and side scatter of the different leukocyte populations (Fig 4A). Monocytes were defined by staining with CD14 and lack of staining with CD3 and CD19 lineage markers (Fig 4B). B lymphocytes were defined by staining with CD19 and lack of staining with CD3 and CD14 antibodies (Fig 4C left hand panel). Natural killer (NK) lymphocytes were defined by staining with CD56 and lack of staining with CD3, CD14 and CD19 antibodies (Fig 4C, centre panel). T lymphocytes were distinguished by staining with anti-CD3 and by their lack of staining with anti-CD14 and anti-CD19 antibodies (Fig 4C, right hand panel). Significant LILRB5 expression was only detected on the surface of CD14+ monocyte cells (Fig 4B). We did not detect significant expression of LILRB5 by B, NK, and T lymphocytes (Fig 4C).

In previous studies we and others have shown that B27 dimer tetramers stain peripheral CD14+ monocytes [9, 21]. B27 dimer staining of CD14 monocytes is inhibited by the anti class I heavy chain (HC10) and anti-LILRB2 MAbs [10]. We next determined whether anti-LILRB5 antisera could inhibit B27 dimer tetramer staining of peripheral monocytes. Consistent with previous published results, B27 dimer tetramers stained peripheral CD14+ monocyte cells strongly ([9, 21] Fig 4D). Normal goat antiserum did not affect B27 dimer tetramer staining of monocytes. Compared to normal goat antiserum, anti-LILRB5 antiserum inhibited B27 dimer tetramer staining of monocytes (Fig 4D).

### LILRB5 but not LILRA5 co-immunoprecipitates with class I heavy chains in transduced cell lines

Lentiviral expression cassettes for expression of N terminal FLAG and C-terminal eGFP tagged LILRA5 and LILRB5 were generated for transduction of the different cell lines in this study. Expression of LILRA5 and LILRB5 fusion proteins was investigated by monitoring eGFP expression by flow cytometry and surface expression by staining with anti-FLAG MAb. Fusion protein expression was also measured biochemically by western blotting with anti FLAG MAb.

**Fig 5:**
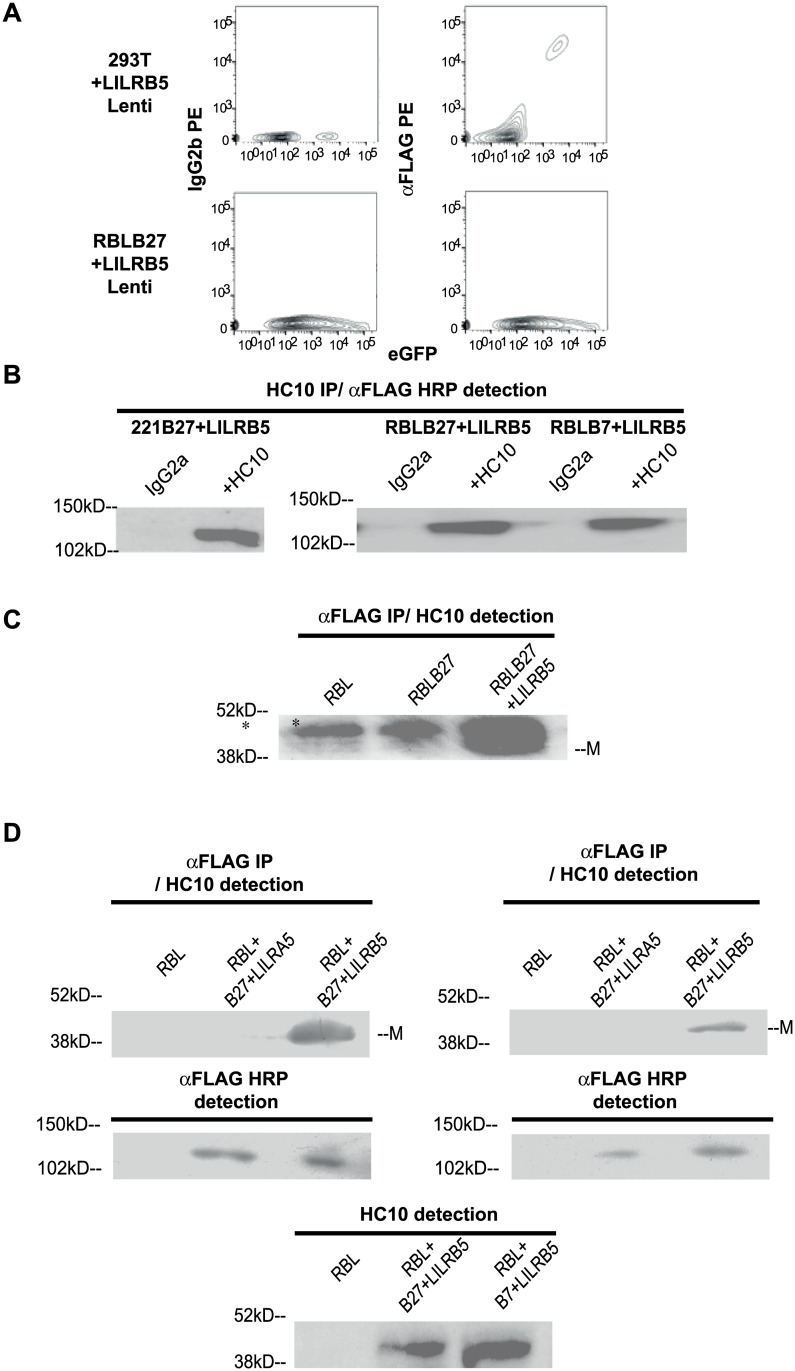
LILRB5 binds to HLA-B7 and HLA-B27 free heavy chains. **A.** Isotype control (IgG2b), anti-FLAG (αFLAG) and eGFP staining of 293T (upper panels) and RBL cells (lower panels) transduced with LILRB5 lentivirus. **B** Left hand panel. Representative western blot of immunoprecipitates from cell lysates of 221B27 cells transduced with LILRB5 with isotype control MAb (IgG2a) or the class I heavy chain MAb HC10. Blots were probed with HRP conjugated anti-FLAG MAb. Right hand panel. Representative western blot of immunoprecipitates from cell lysates of RBLB27 and RBLB7 cells transduced with LILRB5 with isotype control MAb (IgG2a) or the class I heavy chain MAb HC10. Blots were probed with HRP conjugated anti-FLAG MAb. **C.** Upper panel. Representative western blot of immunoprecipitates with rabbit anti-FLAG antisera from cell lysates from parental RBL or RBL cells transduced with B27 and RBL cells transduced with LILRB5 and HLA-B27. Western blots were probed with the class I heavy chain MAb HC10 and HRP-conjugated-anti-mouse Igs. The asterisked band is non-specific and the position of monomeric class I heavy chain (M) is indicated. **D.** Upper panels. Representative western blots of anti-FLAG immunoprecipitates or cell lysates from RBLB27 and RBLB7 cells transduced with LILRA5 or LILRB5. Western blots of immunoprecipitates were probed with the class I heavy chain MAb HC10 and HRP-conjugated-anti-mouse Igs. Centre panels. Western blots of cell lysates were probed with HRP-conjugated mouse anti-FLAG antibody as indicated. Lower panel. Representative western blot of with HC10 MAb of cell lysates from RBL cells or RBL cells transduced with LILRB5 and HLA-B27 or HLA-B7. Representative blots from 1 of 2 independent experiments. All immunoprecipitates were resolved by SDS-PAGE performed under reducing conditions.

B lymphoblasts and mast cells have been shown to express mRNA for LILRB5. Thus, we transduced the human B lymphoblast cell line LCL.721.221 and the rat basophil RBL line with LILRB5 lentivirus. We also transduced 293T cells with LILRB5. Surface expression of LILRB5 was assessed by staining with anti-FLAG antibody. Although LILRB5 was expressed at the cell surface of transduced 293T cells (Fig 5A), we did not detect cell surface expression of LILRB5 by staining transduced RBL or 221 cells with anti-FLAG antibody (Fig 5A and results not shown). This was despite high levels of expression of LILRB5 in these cells as assessed by western blotting of immunoprecipitates and eGFP expression by flow cytometry (Fig 5A and 5B and results not shown).

We next determined whether FLAG-tagged LILRB5 could bind to HLA-class I heavy chains in transduced cells by performing coimmunoprecipitations with anti HLA-class I heavy chain MAb HC10 or anti-FLAG MAb. Immunoprecipitates were resolved by reducing SDS-PAGE. Subsequently LILRB5 binding to class I heavy chain was detected by western blots with HC10 or anti-FLAG antibodies. LILRB5 was detectable by western blot when HLA-class I heavy chains from RBL, 221 and 220 cell lysates coexpressing LILRB5 and HLA-class I were immunoprecipitated with HC10 MAb. Immunoprecipitates were resolved by SDS PAGE and subsequently a band of molecular weight 120kD corresponding in size to eGFP and FLAG-tagged LILRB5 was detected when western blots were probed with anti-FLAG MAb (Fig 5B, left and right hand panels). Conversely, bands corresponding in molecular weight to monomeric HLA-class I heavy chains were identified by western blots probed with HC10 MAb with LILRB5 immunoprecipitates with rabbit anti-FLAG polyclonal antiserum (Fig 5C). No bands corresponding to HLA-class I heavy chains were detected in immunoprecipitates with anti-FLAG antiserum from RBL cells or HLA-B27 expressing RBL cells (Fig 5C). No bands corresponding to HLA-class I heavy chains were detected in immunoprecipitates with anti-FLAG antibody from HLA-B27 or HLA-B7 expressing RBL cells transduced with LILRA5 (Fig 5D upper panels). Transduced HLA-B27 and HLA-B7 RBL cells expressed LILRA5 and LILRB5 which was detected on western blots of cell lysates probed with anti-FLAG mAb (Fig 5D centre panels).

## Conclusions

Here we show that the leukocyte immunoglobulin-like receptor LILRB5 binds to HLA-B27 and HLA-B7 heavy chains. LILRB5 is an innate immune receptor from the Leukocyte Immunoglobulin-like Receptor (LILR) family. LILRB5 is a typical type-1 transmembrane protein incorporating 4 extracellular immunoglobulin superfamily (IGSF) domains. Like other LILRB family members, LILRB5 possesses intracellular tyrosine-based inhibitory motifs (ITIMs) in the cytoplasmic domain. Several other LILRB subfamily receptors are expressed on immune cells where they bind to MHC class I molecules on antigen-presenting cells and regulate immune responses.

LILRB5 transcripts have been detected in natural killer cells, B lymphoblasts, mast cells, monocytes, macrophages and osteoclasts [3–5]. To date, only mast cells have been shown to express LILRB5 protein. In mast cells LILRB5 is expressed intracellularly and secreted upon degranulation. Here we show in addition, by staining with antiserum, that LILRB5 is expressed on the surface of peripheral monocytes.

LILRB2 has previously been shown to bind to both β2m-associated and β2m-free HLA-class I heavy chain forms. The D1 and D2 domains of LILRB2 are involved in binding to both β2m-associated and β2m-free HLA-class I molecules [7–10]. Our studies with tetramer staining and coimmunoprecipitations show that LILRB is distinct in its binding properties from LILRB2, binding to HLA-B27 heavy chain dimers and other HLA class I heavy chains. The distinct binding characteristics of LILRB5 are highlighted by the differences in potential contact residues of the D1 and D2 HLA-class I ligand binding domains of LILRB5, LILRA1, LILRB1 and LILRB2.

Based on structural and sequence similarities the LILR family has been subdivided into type I receptors including LILRB1, LILRB2, LILRA1 and A3 which bind to or which are predicted to bind to HLA-class I [25–27]. Of these receptors, LILRA3 lacks a transmembrane region and is thought to be primarily expressed as a secreted protein [23]. LILRB1 binds to β2m-associated HLA-class I [28]. By contrast LILRA1, LILRA3 and LILRB2 bind to both β2m-associated HLA-class I and β2m-free HLA-class I heavy chains [23, 28]. LILRB3 and LILRB4 do not bind HLA-class I [1, 9, 29]. Type II receptors which include LILRB5 are predicted not to bind to HLA class I. Here we show, LILRB5 is distinct in its binding properties from LILRB2 and LILRA1 and A3, binding to HLA-B27 heavy chain dimers and other HLA class I heavy chains. The distinct binding characteristics of LILRB5 are highlighted by the differences in potential contact residues of the D1 and D2 ligand binding domains of LILRB5 and LILRB1 and LILRB2 with HLA-class I. Our results suggest that unique properties of β2m-free heavy chain forms of HLA-class I could enable binding to the LILRB5 receptor. Class I heavy chains bound specifically to LILRB5 since B27 FHC dimers did not bind to LILRA4, A5 and A6. We tested the ability of LILRB5 antisera to block B27 dimer binding. LILRB5 antisera stained LILRB5 but did not stain LILRA1, A4, A5 and A6 or LILRB1 and B2 transfectants in this study. LILRB5 antisera inhibited B27 free heavy chain dimer binding to LILRB5 transfectants, while not affecting B27 dimer binding to LILRA1 and LILRB2 transfected cells. Moreover B27 dimer tetramer binding to cells transfected with LILRB5 could be blocked with the class I heavy chain MAb HC10 and anti-LILRB5 antisera. B27 dimer staining of peripheral monocytes which express physiological levels of LILRB5 was also inhibited by LILRB5 antisera.

It was previously proposed that the crucial residues that determine binding of Group 1 LILRs to β2m-associated HLA class I are Lys42 and Lys43 (KK), Ile47 and Thr48 (IT), and Leu54 and Val55 (LV) in the D1 domain. The hydrophobic 3_10_ helices formed by these residues orient the key residues for binding to β2m-associated HLA-class I [24, 30].

Co-immunoprecipitations with HLA-class I heavy chain or anti-FLAG antibodies also showed that LILRB5 can bind to HLA-B7 and HLA-B27 heavy chains in cells. There have been no studies on the possible intracellular role of LILR molecules. It is possible that some LILR such as LILRB5 could not only bind to HLA-class I at the cell membrane but also regulate HLA class I expression inside the cell.

Among the LILR family LILRA1 and A3 and LILRB1 and B2 have previously been shown to bind to HLA-class I molecules. LILRB2 has been reported to bind both to HLA-class I on the same cell in a *cis* configuration and with HLA class I on different cells in a *trans* configuration. LILRB2 binding to HLA-class I in the *cis* configuration inhibits mast cell degranulation [11]. Our biochemical and flow cytometry results are consistent with possible *cis* binding of LILRB5 to HLA-class I heavy chains in an intracellular compartment. Our observations of LILRB5 binding to HLA-class I heavy chains suggest a possible regulatory for this protein in HLA-class I assembly and turnover.

Here we show that the leukocyte immunoglobulin-like receptor family member LILRB5 binds to HLA-B7 and B27 heavy chains at the cell surface and intracellularly. Thus, in addition to their binding to HLA-class I and other ligands at the cell surface it is possible that some LILR molecules such as LILRB5 could play a role in regulating class I expression by interacting with HLA-class I molecules within the cell.

## Supporting Information

S1 Table Primer sequences for generation of lentiviral and eGFP and FLAG-tagged expression constructs used in this study. (EPS)

S1 Fig Sequence alignments of the D1 and D2 binding domains of LILRB1 and LILRB2 with the corresponding domains in LILRB5 and LILRA4, A5 and A6. **A** Sequence alignment was performed by Multalin (Version 5.4.1). Gaps (dashes) were introduced to maximize homologies. Amino acids identical to the consensus are indicated in red. Contact residues for LILRB1 and LILRB2 with β2m-associated HLA-class I ligands are marked with a hatch or asterisk respectively. Arrows indicate the positions of β strands. The start of the D1, Ig-like extracellular domain 1, and D2, Ig-like extracellular domain 2 are indicated.
(EPS)
